# Single‐cell sequencing: Current applications in various tuberculosis specimen types

**DOI:** 10.1111/cpr.13698

**Published:** 2024-07-02

**Authors:** Yuqin Zeng, Quan Ma, Jinyun Chen, Xingxing Kong, Zhanpeng Chen, Huazhen Liu, Lanlan Liu, Yan Qian, Xiaomin Wang, Shuihua Lu

**Affiliations:** ^1^ National Clinical Research Center for Infectious Disease Shenzhen Third People's Hospital Shenzhen Guangdong Province China

## Abstract

Tuberculosis (TB) is a chronic disease caused by *Mycobacterium tuberculosis* (*M.tb*) and responsible for millions of deaths worldwide each year. It has a complex pathogenesis that primarily affects the lungs but can also impact systemic organs. In recent years, single‐cell sequencing technology has been utilized to characterize the composition and proportion of immune cell subpopulations associated with the pathogenesis of TB disease since it has a high resolution that surpasses conventional techniques. This paper reviews the current use of single‐cell sequencing technologies in TB research and their application in analysing specimens from various sources of TB, primarily peripheral blood and lung specimens. The focus is on how these technologies can reveal dynamic changes in immune cell subpopulations, genes and proteins during disease progression after *M.tb* infection. Based on the current findings, single‐cell sequencing has significant potential clinical value in the field of TB research. Next, we will focus on the real‐world applications of the potential targets identified through single‐cell sequencing for diagnostics, therapeutics and the development of effective vaccines.

## Graphical abstract

This paper reviews the current use of single‐cell sequencing technologies in TB research and their application in analysing specimens from various sources of TB. The focus is on how these technologies can reveal dynamic changes in immune cell subpopulations, genes and proteins during disease progression after *M.tb* infection.

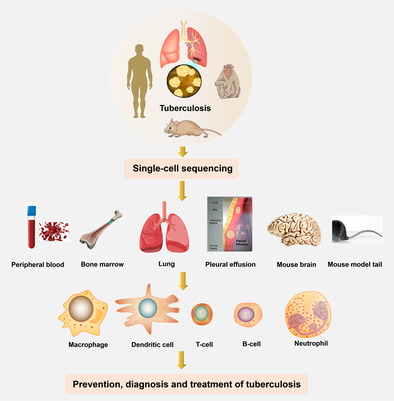

## 1 INTRODUCTION

Tuberculosis (TB) is an infectious disease caused by *Mycobacterium tuberculosis* (*M.tb*) which mainly affects the lungs, resulting in pulmonary tuberculosis, but can also affect any part of the body. *M.tb* infection leads to the development of inflammatory infiltrates, tuberculous nodules, caseous lesions and cavernous formations.^1^ In 2022, a global total of 7.5 million people were newly diagnosed with TB, and approximately 1.3 million people died from TB, which almost doubled the number of deaths caused by HIV/AIDS. Despite being preventable and usually curable, TB remained the world's 13th leading cause of death and the second leading infectious killer after COVID‐19. TB is a significant public health problem and a serious threat to human health.^2^, ^3^

Progress in TB control has been hindered by the rising prevalence of multidrug‐resistant TB (MDR‐TB) and extensively drug‐resistant TB (XDR‐TB),^4^ as well as the delayed diagnosis and misdiagnosis of TB, and the limited availability of effective vaccines. Addressing these issues is crucial for achieving the objective of ending the global TB epidemic by 2035. There is an urgent requirement for accurate, affordable and precise diagnostic tools to detect early TB infection. Additionally, there is a continued need for effective, short and easy‐to‐administer treatment using fewer toxic drugs that are suitable for both drug‐sensitive and drug‐resistant TB patients. Furthermore, the development of new TB vaccines more effective than BCG is essential for TB prevention.^5^

WHO statistics show that only 58% of TB patients in China had bacteriologic laboratory evidence in 2021, which is lower than the global average,^3^ while the diagnosis of the rest of TB patients mainly relies on clinical manifestations and imaging evidence, which lacks specificity. Sputum smear and culture are the most commonly used laboratory diagnostic techniques for TB, but their low sensitivity greatly limits the TB positive rate.^6^ In recent years, molecular biology assays such as high‐throughput sequencing (HTS) have been used increasingly in clinical specimens to identify *M.tb* and provide information on drug susceptibility, and can also differentiate between relapsed infection and reinfection.^7^ Drug‐resistant tuberculosis refers to the phenomenon in which *M.tb* in certain patients has developed resistance to drugs, leading to a decrease in the effectiveness of anti‐tuberculosis drugs and impacting the therapeutic outcome.^8^ The traditional drug resistance test involves observing the growth inhibition of *M.tb* in the drug‐containing media to differentiate between drug‐sensitive and drug‐resistant bacteria. This is typically done using methods such as the proportion method, drug‐resistant proportion method and absolute concentration method. However, these methods are known to be time‐consuming, labour‐intensive and complex to perform.^9^

HTS is a revolutionary change from the traditional Sanger sequencing technology. It has the capability to sequence hundreds of thousands to millions of nucleic acid molecules simultaneously, and thus is known as Next Generation Sequencing (NGS). The emergence of HTS has made it possible to analyse the transcriptomes and genomes of species in a detailed and comprehensive manner. With the rapid development of NGS technology, whole‐genome sequencing (WGS) analysis of *M.tb* have demonstrated their high‐resolution advantages in identifying drug resistance and evolution, analysing transmission patterns and studying genetic heterogeneity.^10^ However, traditional bulk sequencing is generally based on NGS and relies on tissue‐level or population cells. It is not combined with microfluidics, which can only capture the average signals from multiple cells.^11^ As a result, traditional bulk sequencing fails to provide signals demonstrated by specific cell populations or cell states, leading to a significant loss of information regarding cellular heterogeneity.^12^ Numerous studies have demonstrated that the immune response to TB infection typically relies on sequential cell state transitions from the inactive to the active‐like state which can elucidate the nature and potential interactions of the immune cell activation process, contributing to identification of crucial genes and revealing novel regulatory mechanisms.^13^, ^14^

Single‐cell sequencing technology refers to the sequencing of a single‐cell genome or transcriptome in order to elucidate genomic, transcriptomic or other multi‐omics information to reveal the inherent properties of a single cell from the large scale of the genome, the heterogeneity in cellular populations and the cellular maps. Since the development of the first single‐cell transcriptome sequencing technology in 2009,^15^ this technology is increasingly being used in various fields such as oncology,^16^, ^17^, ^18^ immunology,^19^, ^20^, ^21^ neurology,^22^, ^23^ microbiology,^24^, ^25^ reproductive and embryonic medicine,^26^, ^27^ and digestive and urological systems.^28^, ^29^, ^30^ The study of natural killer (NK) cells, dendritic cells (DC) cells, T cells and other immune cells, as well as the analysis of the factors contributing to the heterogeneity of immune cells, are closely related to TB research.^12^

In this perspective, we focus on recent advances in single‐cell sequencing technologies relevant to TB research. These advances primarily involve single‐cell RNA sequencing (scRNA‐seq), single‐cell T‐cell receptor sequencing (scTCR‐seq), cellular indexing of transcriptomes and epitopes by sequencing (CITE‐seq) and assay for transposase‐accessible chromatin with high‐throughput sequencing (ATAC‐seq). Based on the mechanistic process of *M.tb* infection and the diversity of organs involved, we highlight the progress in the application of single‐cell sequencing technology in different TB specimens. This technology is recognized as an essential tool for studying heterogeneity and intercellular dynamics. It also helps to expand the significance and subsequent impact of identifying different cellular phenotypes in the organs affected by TB.

## 2 SINGLE‐CELL SEQUENCING TECHNOLOGY FOR TB RESEARCH

**FIGURE 1:**
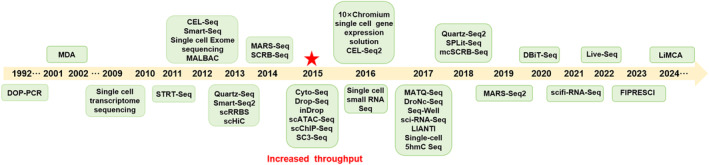
Important timeline of single‐cell sequencing milestones.

**FIGURE 2:**
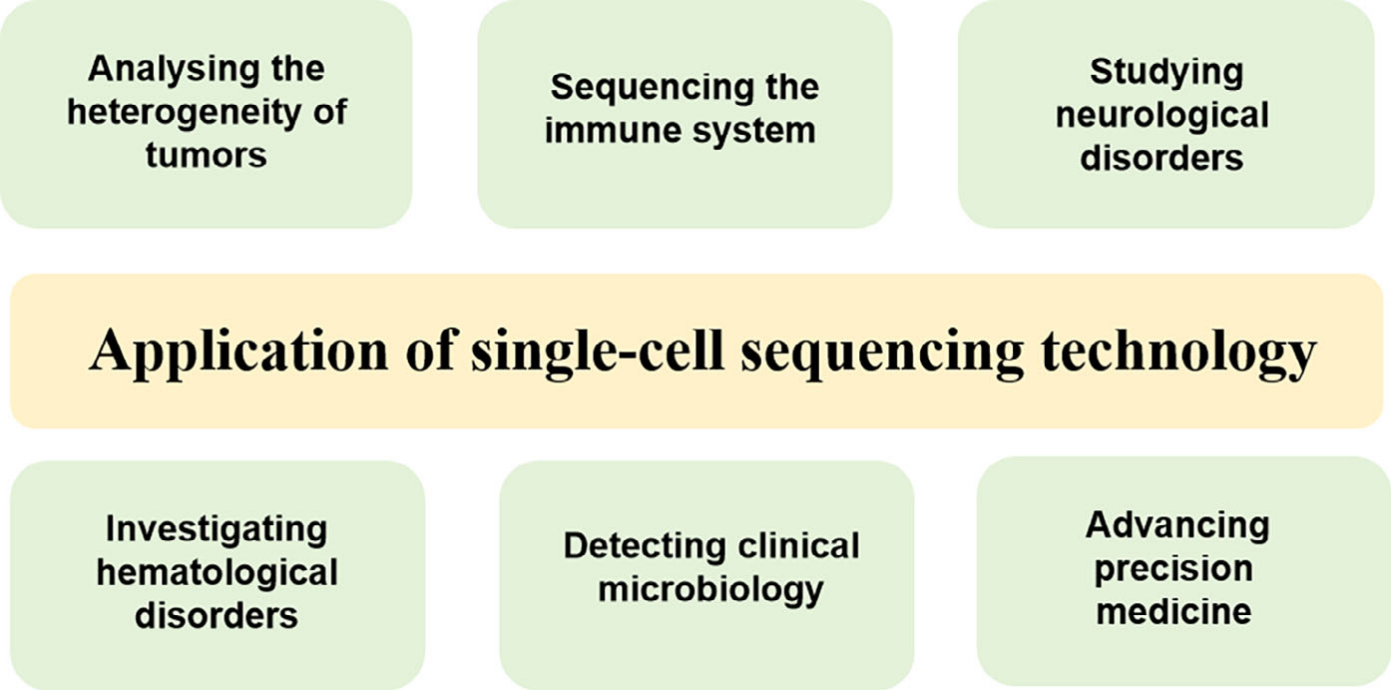
Application areas related to single‐cell sequencing technology.

In recent years, single‐cell sequencing technology has seen significant advancements. Initially limited to analysing only a few cells due to costly isolation techniques, by 2015, high‐throughput methods allowed for the simultaneous analysis of thousands of single cells, and a variety of single‐cell sequencing technologies have been derived (Figure 1). This breakthrough made it practical for clinical applications (Figure 2).^15^, ^31^, ^32^, ^33^, ^34^, ^35^, ^36^, ^37^, ^38^, ^39^ It is noteworthy that an increasing number of researchers have utilized single‐cell sequencing technologies such as scRNA‐seq, TCR‐Seq, CITE‐seq and ATAC‐seq in TB research.

By isolating individual cells, capturing their transcripts, generating sequencing libraries and mapping the transcripts of individual cells, scRNA‐seq offers a comprehensive exploration of cellular diversity, transcriptional profiles and dynamics. Widely applied in life science,^40^, ^41^ such as cell development, immune regulation, oncology, neurobiology and microbiology, COVID‐19,^42^ clinical diagnostics, drug discovery^43^ and TB,^44^ this technology enables the construction of single‐cell maps for tissues, organs and organisms. Researchers have endeavoured to establish whole‐body single‐cell atlases across various species, including *Caenorhabditis elegans*, zebrafish, *Macaca fascicularis*, planarian, *Drosophila melanogaster*, mouse and human beings,^45^, ^46^, ^47^, ^48^, ^49^, ^50^ among others.

T cells and their T‐cell receptor (TCR) repertoire are crucial for immune responses and immunotherapy.^51^ However, the diverse nature of each individual's TCR repertoire poses challenges for traditional identification methods. There is an urgent demand for a rapid, efficient and precise approach to investigate TCR diversity. TCR‐Seq technology facilitates accurate and simultaneous analysis of TCR repertoires on a large scale, with a wealth of publicly available TCR‐Seq data. Sequencing of individual T cells can provide valuable information on the diversity of clonotypes at the single‐cell level. Additionally, it allows the direct analysis and study of the α‐β chain pairing information.^52^ scTCR‐Seq has major applications in (1) assessing antigen‐driven clonal expansion and T cell responses; (2) monitoring the dynamics and heterogeneity of T cell responses longitudinally; and (3) providing relevant information on T cell differentiation pathways and T cell selection through the combined evaluation of TCRs and cellular phenotypes in individual cells. This information not only contributes to the understanding of the aetiology of immune‐related diseases but can also be used to design therapeutic targets.^53^

CITE‐seq integrates gene expression and cell surface protein data at the single‐cell level through sequencing, allowing for the detection of both cell surface proteins and intracellular transcriptome information. By employing DNA barcoding antibodies, CITE‐seq converts protein assays into quantitative sequencing reads. In this technique, antibody‐conjugated oligonucleotides act as synthetic transcripts, which are captured in most large‐scale oligo T‐based scRNA‐seq library preparation methods.^54^ Unlike conventional single‐cell transcriptome sequencing, CITE‐seq provides insight into cell surface protein expression (note: it does not detect intracellular proteins). By combining transcriptomic data within individual cells, this approach enhances understanding of cellular diversity, facilitates precise identification of specific cell types and enables exploration of mechanisms underlying biological processes like therapeutic resistance.^55^, ^56^, ^57^

ATAC‐seq initially inserts DNA transposons into accessible chromatin regions using the Tn5 transposase.^58^ The transposase, carrying these junctions, enters the nucleus and cleaves naked DNA sequences within the open chromatin regions, integrating the junctions into these sites. Finally, NGS is conducted using sequencing tags with known sequences, facilitating the acquisition of the DNA library sequence from the open chromatin regions.^59^, ^60^ Moreover, the development of single‐cell ATAC‐seq (scATAC‐seq) technology has enabled the exploration of epigenomic landscapes at the single‐cell level.^61^ This technique has proven effective in identifying diverse cell types, elucidating regulatory mechanisms of cellular diversity, mapping disease‐associated regulatory elements and reconstructing differentiation pathways.^45^, ^62^, ^63^, ^64^

## 3 CURRENT STATUS OF SINGLE‐CELL SEQUENCING IN DIFFERENT TB SPECIMEN TYPES

### 3.1 Single‐cell sequencing offers great advantages in delving into TB pathogenesis

**FIGURE 3:**
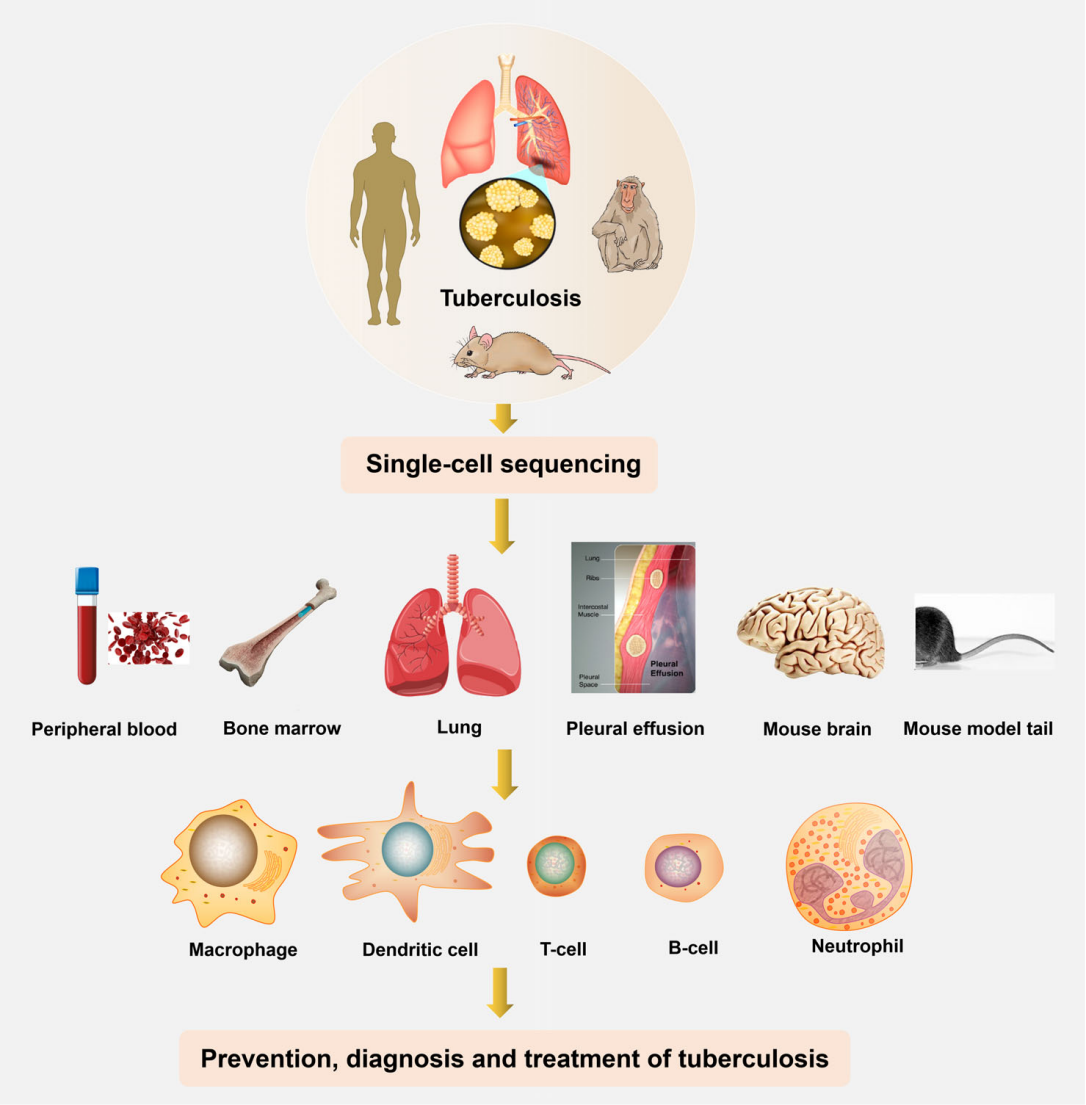
General overview of TB research findings in different specimens using single‐cell sequencing.

Aerosolized droplets containing *M.tb* produced through coughing, sneezing or other open‐mouth behaviours by patients with active TB, are widely spread in the air, they can be inhaled by a new host. These droplets then travel through the oral or nasal cavity, the upper respiratory tract, the bronchial tubes and eventually reach the lungs.^65^, ^66^ The proper functioning of the immune system plays an important role in fighting against TB infection. The immune response is divided into humoral immunity and cellular immune response. *M.tb* primarily infects innate immune cells patrolling the lung, making individuals with compromised cellular immunity more susceptible to TB. When macrophages fail to inhibit or destroy *M.tb*, *M.tb* proliferates within the cell and is released upon macrophage death. A portion of the released *M.tb* spreads via the bloodstream or lymphatic pathway to any body tissue or organ, including highly susceptible areas of TB infection such as the lungs,^67^ throat, lymph nodes, bones, pleura,^68^ spine or kidneys.^69^, ^70^ Another portion of the released *M.tb* may be phagocytosed by other alveolar macrophages. In addition to the cyclic processes described above, lymphocytes and other cells are recruited to the site of infection to initiate a cell‐mediated immune response, which isolate *M.tb* and restrict its further proliferation. The granuloma, which is a compact aggregate of immune cells that consists of macrophages, lymphocytes and other host immune cells, is an important pathological hallmark of *M.tb* infection. A host infected with *M.tb* that is asymptomatic may be because the *M.tb* has been completely eliminated or shield in granuloma, at which point the host enters a state of latent tuberculosis infection (LTBI).^71^, ^72^ As the immune system fails to keep *M.tb* under control, it proliferates rapidly, leading to the reactivation of TB and the development of active TB. At the same time, it also has the ability to spread widely, which is very detrimental to the elimination of TB.^73^, ^74^ Overall, *M.tb* can employ a variety of strategies to evade clearance by the host's innate and adaptive immune responses. These strategies include inhibiting macrophage apoptosis and camouflaging self‐antigens to avoid detection by *M.tb*. The study of the pathogenesis of TB continues to face serious challenges. However, traditional molecular biology techniques and immunological techniques have very limited application in studying, as well as changes in immune cell dynamics caused by *M.tb*‐infected in the lungs and extrapulmonary organs. And also, the implementation of some of these techniques is limited by the type and collection of host specimens. Nevertheless, in recent years, scholars have continued to explore new mechanisms of TB pathogenesis using histologic and epigenetic techniques. Single‐cell sequencing technology is uniquely positioned to study individual cell heterogeneity and intercellular dynamic changes, as well as to identify immune cell subpopulations. It enables the exploration of changes in intercellular communication, gene expression and protein expression following *M.tb* infection. This technology can be applied to specimens such as host peripheral blood, pleural effusion, bronchoalveolar lavage and cerebrospinal fluid (CSF), making it feasible for collection and analysis (Figure 3, Table 1). The development and application of technology have greatly enhanced the study of TB pathogenesis. This advancement has enabled a more comprehensive understanding of the aetiology and pathogenesis of TB, hereby contributing to the prevention, diagnosis and treatment of TB.

**TABLE 1:** Summarizing the most important findings by single‐cell sequencing in different TB specimen types.

Specimen name	Specimen origin/study's subject	Technique	Key cell subpopulations, genes, factors and proteins	Observation	Results to be further validated	Reference
Diverse single‐cell sequencing analysis in peripheral blood for tuberculosis research						
PBMC	Human	scRNA‐seq	Macrophages	A portable, low‐cost scRNA‐seq method called Seq‐Well was developed	(1) Whether the identified cell populations represent different subtypes in PBMCs and *M.tb*‐exposed macrophages. (2) Whether the results of the analysis of differential gene expression correlate with gene sets previously published	81
	Human	SELECT‐seq (scRNA‐seq)	CD8^+^ T cell	Select sequencing of clonally expanded CD8^+^ T cells reveals limits to clonal expansion	(1) Whether expanded CD8^+^ T cells selected by the SELECT‐seq method represent a specific pathogen response. (2) Whether transcriptomic data can reveal the function and characterization of these expanded T cells	82
	Human	scRNA‐seq	NK cell subpopulations (CD3^−^CD7^+^GZMB^+^)	A depletion of a subpopulation of NK cells was revealed in TB	Diagnostic value of the CD3^−^CD7^+^GZMB^+^ subpopulation in differentiating TB from LTBI and HC and changes in the frequency of the CD3^−^CD7^+^GZMB^+^ subpopulation after anti‐TB therapy	84
	Human	scRNA‐seq	CD4^+^ and CD8^+^T cell	Different characterized genes and signalling pathways of depleted CD4^+^ and CD8^+^ T cells were revealed	(1) Whether transcriptomic characterization of CD4 and CD8 T‐cell subsets is consistent across TB patients. (2) Whether connectivity between cell subpopulations shows a similar pattern across TB patients. (3) Whether the findings can be validated and replicated in a larger sample group	85
	Human	scRNA‐seq	CD14^+^ monocyte subpopulation	Proposed association of CD14^+^ monocytes with TB progression	(1) Protective effects of different CD14^+^CD16^+^ monocyte subsets and their frequency and phenotypic changes in treated, vaccinated and other TB patients. (2) Functional role of haemocyte subpopulations and the presence and role of DC subpopulations in CD14^+^CD16^+^ monocytes	87
	Human	scTCR‐seq	T cell	Protective *M.tb* antigen‐specific T cells and antigens associated with infection control were identified	(1) Whether the effect of TB‐specific TCR clusters is consistent across cohorts. (2) Whether the identification of TB‐specific TCR clusters is affected by clustering algorithms	88
	Human	scRNA‐seq, scTCR‐seq	CD8^+^ cytotoxic T cell clusters expressing GZMK, CD4^+^ central memory T cell clusters expressing SOX4, proliferating CD3^+^ T cell clusters expressing MKI67	T cell subpopulations associated with control of TB transmission were identified	(1) Whether CD8^+^ T cell subsets expressing GZMK are associated with the control of TB transmission, and whether CD8^+^/CD4^+^ T cell subsets expressing GZMB and CD4^+^ T cell subsets expressing GZMA are associated with the disease status of TB infection. (2) Functional role of GZMS and potential role of SOX4 in anti‐TB immunization	89
	Human	CITE‐seq, scRNA‐seq	Th17 subpopulations (CD4^+^CD45RO^+^CD26^+^CD161^+^CCR6^+^)	Multimodally profiling memory T cells from a TB cohort identifies cell state associations with demographics, environment and disease	(1) Comprehensive elucidation of differences in memory T cells associated with progression beyond disease‐induced inflammation. (2) Further investigation of unperturbed memory T cell phenotypes	90
	Human	scRNA‐seq， T‐cell/B‐cell receptor (TCR/BCR) sequencing	Monocyte, Th1, CD8^+^T cell, NK cell	Comprehensive immune mapping of the pathogenesis of severe TB patients is depicted	Further studies on the mechanisms of lymphocytopenia in severe TB	91
	Human	scRNA‐seq	*ADM*	Description of *ADM*, a key immune‐related hub gene highly associated with TB	The role and mechanisms of *ADM* in the pathogenesis of TB	92
	Human	scRNA‐seq	Ev‐related differentially expressed genes (DEGs)	Identification of multiple Ev‐associated genes that play important roles in the pathogenesis of TB	The roles and mechanisms of CST3, ABCB1, BSG and GRN in the pathogenesis of TB remain to be further verified experimentally and clinically	93
	Human	scRNA‐seq	C1q	Confirmation of C1q origin from monocytes	Whether soluble, secreted C1q or monocyte‐associated C1q is associated with this inhibitory effect on CD8^+^ T cells	94
	Human	scRNA‐seq	Macrophages expressing *FCN1‐* and *SPP1*	Analysed transcriptomic disease risk and diagnostic biomarkers between COVID‐19 and TB	(1) There is an overlap in some cell types and immune responses between COVID‐19 and TB, and the specific effects and mechanisms are yet to be further investigated and validated. (2) Investigating the shared biological pathway between COVID‐19 and TB to determine if it leads to severe clinical manifestations and whether there is a synergistic effect in cases of co‐infection	95
	Human	scRNA‐seq	GM‐CSF (CSF2)‐activated blood dendritic cells (GM‐CSF‐DCs) differentiated human CD4^+^ T cells	A new Tfh1 differentiation pathway was proposed	CD40 may be a novel therapeutic target in the regulation of Tfh and Tfh1 cells, but its specific role and mechanism require further validation	96
	Human	scRNA‐seq	Monocyte subpopulations	Identified the expansion of peripheral blood monocytes as an indicator of HIV‐1‐TB co‐infection	(1) Whether changes in cell subsets are associated with dysregulated immune responses to HIV‐1‐TB coinfection. (2) Specific roles and mechanisms of CD14^+^CD16^+^ monocyte subsets in HIV‐1‐TB co‐infection	98
	Human	scRNA‐seq	PBMC	Revealed the interaction of genetic background dependence, cell type and pathogen exposure time	(1) The exact mechanism by which specific genetic variants contribute to disease. (2) Whether upstream regulators or downstream regulators of certain specific genetic variants behave differently in various populations	99
Single‐cell sequencing analysis of tuberculosis in the bone marrow						
Bone marrow	Human	scRNA‐seq	BMMNCs, bone marrow	Characterized the transcriptome profile of BMMNCs	The common cellular state of T cells and NKT cells, as well as their progression in patients with X‐SCID requires further study	100
	Mouse model	scRNA‐seq	Blood generating stem cells	Heterogeneity of bone marrow haematopoietic response to *M.tb* infection was confirmed	Whether the altered behaviour of many haematopoietic stem and progenitor cell (HSPC) subpopulations is caused by differences in host signalling, bacterial effectors or other processes remains to be further verified	101
Research analysis and application of single‐cell sequencing technology in lung‐related specimens of tuberculosis						
Lung macrophage	Human/Mouse model	scRNA‐seq, ATAC‐seq	Lung macrophage	Functional heterogeneity of alveolar and interstitial macrophages analysed in *M.tb* infection	(1) Further studies will be conducted in individuals with active TB to explore the relationship between macrophage identity, function and disease state. (2) Investigating whether human alveolar macrophages and interstitial macrophages show a response similar to that observed in mouse lungs after in vivo infection with *M.tb*	102
Lung macrophage	Mouse model	CITE‐seq, scRNA‐seq	Lung macrophage	Protocol for multi‐modal scRNA‐seq on *M.tb* infected mouse lungs	Whether the protocol is compatible with the 5’ scRNA‐seq immune repertoire kits from 10× genomics	103
Lung tissue	Macaque	scRNA‐seq	PDCs, IFN‐responsive macrophage populations, activated T‐cell phases, CD27^+^ NK cell subpopulations	Characterization of different immune cell subpopulations in lung specimens from healthy, LTBI and active TB rhesus macaque models	(1) Temporal dynamics in the lungs of PTB rhesus macaques and the early role of pDCs and IFN‐reactive macrophages in the inflammatory response and initiation of TB in the lungs. (2) NK cells are not found within or near the iBALT structures of the LTBI lung. (3) Do Th1 and Th17 cells correctly localize near *M.tb*‐infected macrophages and mediate *M.tb* control	104
Lung tissue	Mouse model	scRNA‐seq	Lung lymphocyte	Discovered that *M.tb* infection promotes the development of type I IFN features in lung lymphocytes	Implications of increased type I IFN characterization in lung lymphocytes for the generation or pathogenesis of protective immune responses during TB progression	105
Lung tissue	Human	scRNA‐seq	Treg, CD8 T cell, Immunosuppressive myeloid cells, conventional DC, plasma cell‐like DC and neutrophils, etc.	First atlas of *M.tb* infected human lung cells was published	Immunosuppressive cell populations were established to prevent excessive inflammation and tissue damage caused by the persistence of unresolved immune deposits. Protective anti‐TB T‐cell responses were indirectly suppressed, a phenomenon that requires validation through additional studies	106
Lung tissue, Bronchoalveolar lavage	Cynomolgus macaque	scRNA‐seq	TCR	Identification and characterization of the TCR repertoire of the cynomolgus macaque	Macfas and Macmul have amplified TCR beta loci compared to humans, possibly due to gene duplication and changes in selective pressure during evolution of the TCR locus, requiring further validation	107
Lung lobe	Mouse model	scRNA‐seq	CD4 T cell	Revealed changes in the transcriptome of vaccine‐naïve and vaccinated CD4 T cells	(1) The role of CXCR3 in tissue homing of pathogen‐specific T cells. (2) To confirm whether IL‐17 induces and maintains this protective memory T‐cell response and to investigate its specific mechanisms. (3) Investigate whether cytotoxic CD4 T cells can confer resistance to TB. (4) In the models mentioned in the article, do the co‐produced cells differ in their ability to protect against classical Th1 or Th17, and which cell types respond to IL‐17 to mediate protection	108
Lung tissue	Mouse model	scRNA‐seq	Lung T and B cell subpopulations	*M.tb* infected mice are resistant to secondary infection with CoV2, with no impact on *M.tb* burden, this resistance is associated with expansion of lung T and B cells	Whether these findings can be extended to other models of TB that more accurately represent the range of human diseases	109
BALF	Human	scRNA‐seq	Lung macrophage	Study of alveolar macrophage characteristics in alveolar lavage fluid from patients with active pulmonary TB	Further study of BALF specimen from TB patients with varying degrees of disease severity	112
BALF	Human	scRNA‐seq	Macrophages, CD8 T cell	Mapped of BALF‐mediated immunity in different diseases	(1) Expression and function of chemokine profiles CXCL5, CCL20 and CCL23 in LTBI increased after *M.tb* stimulation. (2) LTBI‐specific macrophage and T cell function	113
BALF	Rhesus macaques	scRNA‐seq	T cells, macrophages, key myeloid cell subpopulations	Analysis of the protective immune characteristics that emerge after intravenous BCG vaccination	Mechanisms associated with the initiation of localized myeloid cells in the context of *M.tb* infection and vaccination	114
BALF	Mouse model	scRNA‐seq	Lung macrophage	Discovered that lysosomal injury dysregulates the metabolic and immune responses of alveolar macrophage subpopulations	Whether lysosomal protease‐dependent mechanisms can modify organelles other than mitochondria	115
Granulomas	Cynomolgus macaques	scRNA‐seq	13 generic cell types	Revealed cellular correlates of TB control	Matching analysis of an additional time point post‐infection (p.i.), and analysis of lung tissue and granulomas in vaccinated, reinfected and protected animals	117
Granulomas	Zebrafish/Macaque	scRNA‐seq	*Stat6*	Type 2 immune signalling mediated through *stat6* was found to be absolutely essential for epithelialization and granulomas formation	(1) Whether *stat6* deficiency has additional effects on bacterial burden or inflammatory state may mask the granuloma phenotype. (2) Contribution of the *stat6*‐mediated signalling pathway to human tuberculosis in the context of sarcoidosis in the pulmonary environment	118
Granulomas	Cynomolgus macaques	scRNA‐seq	CD8^+^ lymphocyte	Dissected the importance and function of CD8‐expressing immune cell subpopulations in early *M.tb* infection	(1) Further research is needed to determine which specific cell subpopulations contribute to protection. (2) Whether CD8^+^ lymphocytes directly or indirectly regulate the expression of cytotoxic molecules in T cells through alternative mechanisms	119
Single‐cell sequencing analysis of tuberculous pleural effusion						
Pleural fluid	Human	scRNA‐seq, scTCR‐seq	CD4^+^ and CD8^+^ T cell	Identified unique features and changes in CD4^+^ and CD8^+^ cells	Due to the lack of detection of certain VDJ haplotypes of TCRβ on T cells at the protein level, we are also unaware of whether CD4^+^ or CD8^+^ T cells with specific VDJ haplotypes, featuring a TRBV region of TRBV4‐1, constitute a significant source of T cell expansion at the TB locus	122
	Human	scRNA‐seq, scTCR‐seq	CD8 and CD4^+^T cell subpopulations	Localized T‐cell immune landscape in TB was revealed	(1) Further study is needed on the role played by CTL CD4 T cells in TB. (2) Investigating the specific role of CD8 T cells expressing GZMK in tuberculosis pathogenesis	123
	Human	scRNA‐seq	PFMC	Uncovered the unique localized immune landscape of TPEs distinct from TSPEs and MPEs	Specific molecular mechanisms by which CD4 effector cells kill their target cells and their role in combating *M.tb*	124
Analysis and application of single‐cell sequencing technology in the study of brain specimens from tuberculous meningitis						
Brain tissue, CSF	Mouse model	scRNA‐seq	15 cell types including immune cells, neurons, glial cells, etc.	Molecular mapping and changes in different cell types within the whole brain of TBM mice are depicted	In vivo and in vitro loss‐of‐function/enhancement‐of‐function assays are conducted to validate the functional and mechanistic effects on altered cells, biological processes and genes in TBM	125
Brain tissue	Mouse model	scRNA‐seq	mRNAs and miRNAs in macrophages, Microglia, ependymal cells, neurons and vascular smooth muscle cells	Identification of miRNAs enriched for cell types in TBM brain specimens	Whether miRNAs upregulated during TBM are secreted into the cerebrospinal fluid via exosomes	126
*Mycobacterium marinum* infected mouse model tail tissue for single‐cell sequencing						
Tail tissue	Mouse model	scRNA‐seq	Neutrophil	Demonstrated that ESX‐1‐mediated pathogenesis is dependent on neutrophils	(1) Investigating whether monocytes protect against neutrophil‐induced harmful inflammation in *M.tb* infection without impacting bacterial load. (2) Whether iNOS activity is indeed required for the mechanism of monocyte suppression of ESX‐1‐mediated immunopathology	127

### 3.2 Diverse single‐cell sequencing analysis in peripheral blood for TB research

Peripheral blood is the blood that flows and circulates throughout the body and is a crucial element of the body's immune system. It contains erythrocytes, leukocytes and thrombocytes, which are suspended in the plasma and circulate throughout the body via the plasma. The percentage and phenotype of each immune cell subpopulation in peripheral blood can indicate the individual's immune status and their response to immunotherapy, which has demonstrated potential for predicting treatment efficacy and has been extensively utilized in oncology,^75^, ^76^ TB^77^, ^78^ and other related fields. However, immune cells are diverse and interact with each other. In recent years, many researchers have utilized single‐cell sequencing technology to analyse the transcriptome of peripheral blood, mainly peripheral blood mononuclear cells (PBMCs),^79^, ^80^ which has described many of mechanisms and potential therapeutic targets relevant to TB research.

A portable, low‐cost scRNA‐seq method named Seq‐Well was developed in 2017 by Gierahn et al. They not only used Seq‐Well to analyse primary human macrophages exposed to *M.tb*, but also tested the ability of this technique to disaggregate cell populations in complex primary specimens by performing multicolor cellular imaging after loading PBMCs into a triple array. Ultimately, this study identified various cell clusters and their responses to infection at that time. This simple and portable device could make scRNA‐seq widely available.^81^ In recent years, single‐cell sequencing has been increasingly applied to TB in peripheral blood, and the technology is maturing and diversifying. Another study by Huang et al. stimulated PBMCs from asymptomatic subjects infected with *M.tb* and then screened for activated CD8^+^ T cells using the SELECT‐seq technique. SELECT‐seq involves the selection of rare cells based on RNA expression prior to performing deep scRNA‐seq. They analysed over 3000 *M.tb*‐responsive peripheral blood CD8^+^αβ T cells at the single‐cell level and demonstrated a significant correlation between the gene expression profile of conventional CD8^+^ T cells and clone size. They found that highly expanded conventional clones exhibited decreased expression of the interleukin 2 receptor (IL‐2R) and increased indicators of senescence. Thus, it is concluded that this mechanism of limiting clonal expansion could increase the diversity of T cell receptors. The authors suggested that this study may be crucial in achieving a better understanding of the clonality and function of pathogen‐specific T cells.^82^

Single‐cell sequencing technology is more widely used for identifying new cell types, confirming rare cell populations, constructing cell states and mapping cells.^83^ By comparing scRNA‐seq datasets of PBMC isolated from healthy controls (HC), individuals with LTBI and those with active TB, Cai et al. discovered that the subpopulation of NK cells (CD3^−^CD7^+^GZMB^+^) was significantly reduced in TB compared to HC and LTBI. They also confirmed that the frequency of this subpopulation increased after anti‐TB treatment. This study demonstrated that changes in CD3^−^CD7^+^GZMB^+^ frequency can be used to differentiate TB from HC and LTBI.^84^ Another study by Pan et al. analysed scRNA‐seq of CD4^+^ and CD8^+^ T cells isolated from PBMCs of healthy individuals and TB patients. They identified eight subpopulations in CD4^+^ T cells and seven subpopulations in CD8^+^ T cells, respectively. Furthermore, the study elucidated the changes in the transcriptome landscape and characteristics of each subpopulation. More importantly, they revealed different characteristic genes and signalling pathways of depleted CD4^+^ and CD8^+^ T cells, providing insight into the pathogenesis of T cell exhaustion during active *M.tb* infection.^85^ Previous studies have shown that monocytes play an essential role in TB‐specific immune signalling in the bloodstream.^86^ Hillman et al. performed scRNA‐seq on PBMCs from patients with active TB and HC, and revealed that transcriptomic and functional alterations in the intermediate CD14^+^CD16^+^ monocytes, including increased expression of inflammatory and MHC‐II genes. Moreover, these monocytes exhibited an enhanced capacity to activate T cells, suggesting an overall heightened activation of this cell population. This study further demonstrated that different subpopulations of intermediate CD14^+^CD16^+^ monocytes were responsible for each genetic signature, indicating significant functional heterogeneity within this cell population. Finally, they also observed that CD14^+^ monocytes are associated with TB progression as they exhibit transient changes. As described above, these changes were not observed in the same active TB patient at mid‐treatment.^87^ PBMCs from two cohorts of 116 individuals infected with *M.tb*, that is individuals with TB or with controlled infection, were analysed using scTCR‐seq by Musvosvi et al. The study identified protective *M.tb* antigen‐specific T cells and antigens associated with infection control, providing a valuable target for future vaccine development.^88^ Around the same time, Jiang et al. published a study in *Clinical Immunology*, which also identified T cell subsets associated with the control of TB transmission through single‐cell sequencing. The researchers analysed PBMCs and T cells isolated from both TB patients and healthy individuals and found that the CD8^+^ cytotoxic T‐cell clusters expressing GZMK and the CD4^+^ central memory T‐cell clusters expressing SOX4 were depleted compared to HC. On the other hand, the CD3^+^ T‐cell clusters expressing MKI67, which indicates proliferation, were expanded. These findings could potentially contribute to the prevention of TB transmission.^89^ Memory T cells were isolated from PBMCs of patients with progressive TB using CITE‐seq technology in a previous study. Epitopes were measured and single‐cell transcriptomes were generated in order to identify T‐cell states associated with TB progression. The researchers defined 31 cell states in 259 individuals and identified a Th17 subpopulation labelled CD4^+^CD45RO^+^CD26^+^CD161^+^CCR6^+^ with cytotoxic and pro‐inflammatory properties, as defined by multimodality. In addition, they conducted a further analysis of the differential abundance and function of the Th17 subpopulation between progressor and non‐progressor donors. They discovered that this state responded to *M.tb* peptide stimulation in vitro. This study demonstrates the ability to integrate a multimodal single‐cell atlas in order to characterize cellular states relevant to disease and other features.^90^ Based on single‐cell sequencing technology, Wang's group focused on characterizing the complete immune profile of severe TB patients to understand the pathogenesis. By analysing PBMCs from both healthy control individuals and patients with TB of varying severity, researchers have discovered some notable differences. In severe TB patients, the proportion of inflammatory immune cells, such as monocytes, was significantly increased, while the number of various lymphocytes, including NK cells, was significantly decreased. Additionally, the apoptotic pathways of these immune cells were further characterized. The study also revealed significant activation of Th1, CD8^+^ T and NK cells, along with widespread immune exhaustion. CD8^+^ T and NK cells exhibited high cytotoxicity. Monocytes were found to cause systemic up‐regulation of *S100A12* and *TNFSF13B*, which may be responsible for the inflammatory cytokine storm observed in critically ill patients.^91^

Alternatively, there have been direct analyses of gene expression and associated protein sources in TB PBMCs. Researchers have combined array expression profiling and scRNA‐seq to comprehensively characterize *ADM*, the key immune‐related hub gene highly associated with TB. They concluded that *ADM* could serve as a potential candidate marker capable of distinguishing between HC, LTBI and TB.^92^ In a recent study, researchers identified several extracellular vesicle (Ev)‐related genes from PBMCs of TB patients and healthy individuals using scRNA‐seq. These genes play a crucial role in the pathogenesis of TB and can serve as novel biomarkers and therapeutic targets for TB. Furthermore, the researchers predicted five potential TB drugs using the CMap database.^93^ According to scRNA‐seq combined with flow cytometric analysis of PBMCs, serum levels of C1q, a large 460 kDa protein, correlate with TB disease status and monocytes are C1q producers.^94^

In addition, single‐cell sequencing technology has been widely applied to the study of co‐infections involving *M.tb* and other pathogens, providing new avenues for their prevention and diagnosis. Sheerin et al. obtained qualified COVID‐19 gene expression profiles from human whole blood, PBMCs or bronchoalveolar lavage fluid (BALF) using past data. The analysis results suggested that COVID‐19 and immune co‐modulation complicate the progression of TB co‐infection.^95^ Another study related to TB/COVID‐19 infection reported that monocyte–macrophage colony‐stimulating factor (GM‐CSF)‐activated human blood DC cells stimulated the differentiation of human CD4^+^ T cells into Tfh1 cells. scRNA‐seq analysis showed that only DC cells with high levels of CD40 and low levels of ICOS ligands were able to efficiently drive Tfh1 polarization. This polarization profile was closely associated with patients with active TB as well as patients with COVID‐19. It represents a novel Tfh1 differentiation pathway that could serve as a potential therapeutic target.^96^ In addition, co‐infection of *M.tb* and human immunodeficiency virus (HIV) continues to pose a significant healthcare burden. HIV co‐infection during TB predisposes the host to the LTBI, worsening the disease status and increasing mortality rates.^97^ Zhang's group performed scRNA‐seq on PBMCs from patients with uncomplicated HIV‐1 infection (HIV‐pre) and patients who were not receiving anti‐tuberculosis treatment (HIV‐1 pre and TB‐pre), as well as patients receiving anti‐tuberculosis treatment (HIV‐pre and TB‐pos). This study comprehensively mapped the transcriptome of immune cell subpopulations in PBMCs. It not only demonstrated the role of monocyte subpopulations in peripheral blood as diagnostic markers in HIV‐1/*M.tb* co‐infection, but also revealed the corresponding immune dysregulation in different disease states.^98^ There was also a study based on a scRNA‐seq dataset of PBMC (exposed to three pathogens: *M.tb*, *Candida albicans* and *Pseudomonas aeruginosa*) from 120 healthy individuals, which revealed the interaction between genetic background dependence, cell type and duration of exposure.^99^

Overall, with the increasing maturity and sophistication of single‐cell sequencing technology, studies on the peripheral blood of TB origin have become rich and diverse. These studies focus on the dynamic changes of different immune cell subpopulations before and after *M.tb* infection, as well as co‐infection with other pathogens. They also investigate the related gene expression and protein sources. The aforementioned research results provide valuable insights into the progression of TB disease and the identification of new targets for diagnosis and treatment.

### 3.3 Single‐cell sequencing analysis of TB in the bone marrow

Bone marrow differs from peripheral blood in that it has its own specialized blood supply. Currently, there are not many reported applications of single‐cell sequencing‐based technologies related to bone marrow in the field of TB. Dong et al. applied scRNA‐seq to characterize the transcriptome profiles of bone marrow‐derived mononuclear cells (BMMNCs). They found that both maternal and autologous T cells and NK T cells exhibited exhaustion‐like dysfunction in patients with X‐linked severe combined immunodeficiency disease (XL‐SCID) who had relapsed and persistent *M.tb* infection.^100^ Heterogeneity exists in the haematopoietic response of the bone marrow to *M.tb* infection. Researchers analysed haematopoietic stem cells after HN878 *M.tb* and H445Y *M.tb* infections using scRNA‐seq and found that HN878 *M.tb* infection resulted in the expansion of haematopoietic stem cells and enhanced lymphocyte production. Meanwhile, H445Y *M.tb* infection led to an increase in the number of granulocyte‐monocyte progenitors (GMP) and mature granulocytes and monocytes. This study presents an in‐depth view of host–pathogen interactions and provides new ideas for the treatment of TB.^101^

### 3.4 Research analysis and application of single‐cell sequencing technology in lung‐related specimens of TB

By reviewing the published studies related to TB single‐cell sequencing, a significant proportion of lung‐related specimens from models or populations infected with *M.tb*. The lung also plays a crucial role in the process of *M.tb* infection. In a study published in 2021, Pisu et al. present a novel approach that combines bacterial fitness fluorescent reporter strains with scRNA‐seq and ATAC‐seq. This approach was applied to analyse the functional heterogeneity of alveolar and mesenchymal macrophages in *M.tb*‐infected lungs collected from mice. The study implies that single‐cell sequencing technologies are being increasingly applied to the study of lung specimens from humans and animal models. This research further deepened researchers' understanding of the mapping of immune response in the lungs of healthy and *M.tb‐*infected individuals.^102^ Subsequently, in 2023, Davide Pisu and David G. Russell published a protocol that outlines the procedures for isolation, staining (using CITE‐seq antibodies), methanol fixation and generation of scRNA‐seq libraries for *M.tb*‐infected lung macrophages. This protocol can be utilized for mouse, non‐human primate and human‐infected tissues.^103^

Esaulova et al. utilized scRNA‐seq and time‐of‐flight cytometry (CyTOF) to analyse and classify various subgroups of immune cells in lung specimens obtained from healthy rhesus monkey models, as well as those with LTBI and active TB. They concluded that NK cells provide protection during the latent phase, whereas the accumulation of plasmacytoid dendritic cells (pDCs) and interferon‐responsive macrophages, as well as activated T cells, characterize TB disease.^104^ Akter et al. conducted a study where they examined lung lymphocytes from both healthy and *M.tb*‐infected mice using scRNA‐seq. The study revealed that type I interferon (IFN) was found to be enriched in lymphocytes and was accompanied by pulmonary heat shock in NK cells. Additionally, the study verified that locus A (*Ly6A*) was highly expressed on the surface of activated lymphocytes following *M.tb* infection.^105^ The first cellular atlas of human lungs infected with TB was published in 2023. Using healthy tissues from donors in public databases as controls, researchers utilized scRNA‐seq to analyse lung tissues from TB patients at various stages of inflammation. This analysis revealed a diverse range of immune cell subpopulations, including regulatory T cells (Treg), depleted CD8 T cells, immunosuppressive myeloid cells, conventional DC cells, plasma cell‐like DC and neutrophils, among others. In inflamed lungs, immune cells upregulated genes associated with ATP synthesis and interferon‐mediated signalling, indicating a positive immune response to *M.tb* infection. In addition, there was extensive crosstalk between these two types of cells. Notably, the presence of genes associated with TB progression in immunosuppressive myeloid cells and Treg could be a potential target for TB research.^106^ Jaiswal et al. investigated the T‐cell receptor (TCR) expression libraries in lung specimens from the *M.tb*‐infected cynomolgus macaques using scRNA. The study identified and analysed the alpha (TRA), beta (TRB), gamma (TRG) and delta (TRD) loci, and identified functional genes based on their expression. This research improves the cynomolgus macaques model for broader application in the study of human diseases.^107^ Similarly, this could be used to amplify variable region genes in rhesus monkeys. Single‐cell sequencing technology could also be helpful in the development of effective vaccines. In their research, Jong et al. investigated changes in the transcriptome of CD4 T cells in the lungs of *M.tb*‐infected mice before and after CDN vaccination using scRNA‐seq data. They discovered that the metabolism of Th17 cells was enhanced, indicating an activation of effector functions. This suggests that the expression of *Tnfsf8* (CD153) may play a role in vaccine‐induced protection.^108^

The immune response induced by *M.tb* infection is resistant to SARS‐CoV‐2 (CoV2). Resistance in *M.tb* infected mice is associated with the expansion of T and B cell subpopulations in the lungs upon viral challenge were demonstrated through scRNA‐seq by Rosas et al. in 2022.^109^

BALF is unique in providing a bacteriological basis for diagnosing tuberculosis. It directly collects inflammatory immune effector cells in the lungs and detects the values of lymphocyte subpopulations. These values can reflect the local cellular immune function of the lungs, thus considerably increasing the accuracy of TB diagnosis.^110^, ^111^ Chen et al. analysed alveolar macrophages in BALF from three patients with active TB using scRNA‐seq, and compared it with BALF from three healthy individuals from public databases as a control. They found a potential polarization process in the subpopulation of alveolar macrophages, where they transitioned from M1‐type to M2‐type. The proportion of this transition was increased in patients with active TB. Additionally, there was an increase in cell–cell communication and enhancement of alveolar macrophages.^112^ In conclusion, this study confirmed the characterization of alveolar macrophages from BALF of patients with active TB by using scRNA‐seq. Yang et al. analysed the immune profiles of BALF at different stages of TB with the use of scRNA‐seq. In particular, they identified the interactions between monocyte–macrophage (MM)‐CCL‐23 and CD8^−^CDR6 during LTBI, which suggests a potential target for TB control. The discovery of this lung immune profile provides a new scientific foundation for designing effective vaccines and host‐targeted therapies for TB in the future.^113^ Rhesus macaques inoculated intravenously with the BCG vaccine develop a distinct protective immune profile that was characterized by an influx of multifunctional T cells and macrophages into the airways. Also, intercellular immune signalling pathways between key myeloid and T‐cell subpopulations were strengthened under the stimulation of purified protein derivative (PPD).^114^ In another interesting study, Bussi et al. used scRNA‐seq to analyse lung macrophages in a mouse model infected with *M.tb* with endothelial injury. The study demonstrated that lysosomal injury disrupted the metabolic and immune responses of alveolar macrophage subpopulations.^115^

The composition of granulomas varies considerably. Even in a single individual, infection can result in granulomas with varying histologic features. Each granuloma changes independently over time. In a nonhuman primate model of TB infection, each individual had more than 10 granulomas, each of which is very variable in terms of inflammatory characteristics, size and bacterial ecology.^13^, ^116^ Gideon et al. obtained pulmonary granulomas from a cynomolgus macaques model of TB. They characterized the cellular composition and intercellular signalling networks using scRNA‐seq. The study defined the complex multicellular ecosystem of the granulomas and suggested potential targets for host immunity. These findings could be useful for the development of effective vaccines and therapeutics for TB.^117^ Cronan et al. discovered that type 2 immune signalling, mediated through *stat6*, is crucial for epithelialization and granulomas formation. This was determined through the use of scRNA‐seq on granulomas from zebrafish‐*Mycobacterium marinum* model and *M.tb*‐infected macaques model. The study suggests that a comprehensive understanding of the signalling environment that facilitates the formation, structure and dynamics of epithelioid granulomas could open up new therapeutic possibilities for TB treatment.^118^ Subpopulations of immune cells expressing CD8 play an important role in early *M.tb* infection. Researchers performed scRNA‐seq on granulomas of cynomolgus macaques and demonstrated the presence of multifunctional cytotoxic CD8^+^ lymphocytes in control granulomas. Surprisingly, CD8‐depleted animals exhibited an enrichment of CD4 and γδ T cells. This significantly impaired early control of *M.tb* infection, leading to increased formation of granulomas, lung inflammation and bacterial burden.^119^

In summary, single‐cell sequencing technology has advanced significantly in infection models, such as mice, zebrafish and non‐human primates (NHP), as well as in lung tissues, BALF and granulomas of TB patients. This technology provides a more comprehensive understanding of host immune responses and immune cell heterogeneity, contributing to the development of effective vaccines and treatments for TB.

### 3.5 Single‐cell sequencing analysis of TB pleural effusion

Tuberculous pleural effusion (TPE) is one of the most common forms of extrapulmonary tuberculosis and a frequent cause of pleural effusions (PEs) in TB‐endemic regions. It is caused by *M.tb*, which leads to a massive influx of immune cells into the pleural cavity.^120^, ^121^ As a result, single‐cell sequencing is currently being used to analyse the response to *M.tb* infection. Shao et al. analysed T cells in TPE using scTCR‐seq, scRNA‐seq and flow cytometry. They identified unique characteristics and variants of CD4^+^ and CD8^+^ T cells, and discovered a novel multifunctional subpopulation of CD4^+^ T cells. This subpopulation, which exhibits CD1‐restricted, Th1 and cytotoxic features, may indicate a potential immune response to the presence of protective immunity against *M.tb*.^122^ Cai et al. revealed the T‐cell immune landscape of TB at a local level with the use of scRNA‐seq and scTCR‐seq techniques. The researchers analysed PBMCs and pleural fluid mononuclear cells (PFMCs) from 354 patients with TPE. They found that CD8 and CD4^+^ T cell subpopulations exhibit distinct effector functions, which were particularly pronounced at the pleural site. Of particular note, *M.tb* can induce the expression of GZMK protein in CD8 T cells, suggesting that the accumulation of CD8 T cells is a potentially vital feature of *M.tb* infection and is likely to play a role in the pathogenesis of TB. These findings may also have implications for vaccine development.^123^ By performing scRNA‐seq on single nucleated cells in TPE, transudative pleural effusion (TSPE) and malignant pleural effusion (MPE), Yang et al. demonstrated that the characteristics of the immune response differed among the three conditions. Specifically, cell types such as NK cells, CD4^+^ T cells and macrophages were predominantly present in TPE. The CD4 lymphocyte population favoured Th1 and Th17 responses, whereas NK cells exhibited immune exhaustion. Macrophages were responsible for driving the elevation of systemic inflammatory response genes and pro‐inflammatory cytokines. The above results revealed a distinct local immune landscape of TPE, separate from that of TSPE and MPE. This finding deepens our understanding of the immune mechanisms at the infection site and identifies potential targets for therapeutic interventions.^124^

### 3.6 Analysis and application of single‐cell sequencing technology in the study of brain specimens from TB meningitis

Tuberculous meningitis (TBM) is the most severe and fatal manifestation of TB. In recent years, there has been limited research on the application of single‐cell sequencing technology in the field of TBM. However, the analysis of brain tissue from TBM patients is an important and valuable research platform. Zhang et al. conducted a study in which they created a mouse model of TBM using attenuated *M.tb* induction. They then analysed the entire brain tissue using scRNA‐seq, which yielded the identification of 15 different cell types, including immune cells, neurons and glial cells. The study also provided molecular profiles and highlighted changes in different cell types within the brain of TBM mice. Furthermore, the study confirmed that in TBM, the immune response is activated and there are partial metabolic changes.^125^ Zhang's group recently published another study on single‐cell analysis of the TBM mouse model. Once again, the researchers constructed a TBM mouse model and generated single‐cell suspensions of brain tissue for scRNA‐seq. This study involved a thorough investigation of mRNA expression and miRNA activity in different cell types (e.g., macrophages, microglia, ependymal cells, neurons and vascular smooth muscle cells) during the development of TBM. The researchers also identified miRNAs that are abundant in specific cell types in TBM brain specimens.^126^

### 3.7 *Mycobacterium marinum* infected mouse model tail tissue for single‐cell sequencing

In most published studies, single‐cell sequencing analysis of specimens from peripheral blood, lung, PE, brain and other sources from *M.tb* infected mouse models has been widely reported. In contrast, Lienard et al. used scRNA‐seq analysis to examine neutrophils isolated from tail tissues of *M. marinum* infected mice. They demonstrated that ESX‐1‐mediated pathomechanisms are dependent on neutrophils, suggesting that neutrophils have a detrimental effect on the host during *M.tb* and *M. marinum* infection.^127^

## 4 CURRENT RESEARCH LIMITATIONS AND FUTURE RESEARCH DIRECTIONS

Single‐cell sequencing, a rapidly advancing technology in recent years, is a part of the broader trend in the field of TB. In contrast to other traditional research methods, single‐cell sequencing technology has achieved unique accomplishments in the field of TB research. First, it can reveal the expression patterns and functions of different cell types during the tuberculosis infection process, helping researchers gain a better understanding of the heterogeneity among cells. Through single‐cell sequencing, researchers have identified novel immune cell subtypes that play crucial roles in the immune response to TB, facilitating a deeper exploration of the immune response mechanisms. Single‐cell sequencing technology can help researchers understand cellular‐level differences among different patients, providing essential insights for the development of personalized treatment strategies. In summary, the application of single‐cell sequencing in TB research has provided us with a deeper understanding at the cellular level and the potential for personalized treatment.

Although numerous studies on TB by single‐cell sequencing have been conducted using specimens from various sources, there is still a need to further investigate the response of different immune cell subpopulations or related genes to *M.tb* infection. This will help establish a more robust foundation for the prevention, diagnosis and treatment of TB. By summarizing the single‐cell sequencing studies performed in the field of TB in recent years, it is evident that there are still several limitations that need to be addressed in order to achieve the aforementioned goals. First, on the one hand, it is clear single‐cell sequencing technology has been used primarily to analyse cell subpopulations or genes in peripheral blood and lung specimens from TB patients. Conversely, less attention has been given to studying other sites of extrapulmonary TB involvement, such as the thorax and the brain. Although *M.tb* infections are most commonly found in the lungs, and studies related to pulmonary TB are most abundant, the proportion of extrapulmonary TB is also increasing year by year, contributing to the global burden of TB. Therefore, research on extrapulmonary TB is becoming increasingly necessary. Conducting a comprehensive study in these areas could significantly contribute to a better understanding of the pathogenesis of TB. In addition, future studies on single‐cell sequencing may focus on groups of tissue samples to elucidate the immune microenvironment of TB. This will provide a more in‐depth and comprehensive basis for elucidating the pathogenic mechanism of *M.tb*, the course of TB and the development of therapeutic strategies. On the other hand, in order to study the dynamic interactions of TB as a chronic infection, the selection of appropriate clinical samples is crucial. First, whenever possible, samples from the same patient at multiple time points should be collected to observe the dynamics of TB over the course of the disease. Time‐series samples can offer valuable insights into disease progression and the effectiveness of treatments. Second, samples from TB patients with different clinical presentations, such as active TB patients and LTBI, are selected to study immune responses and pathogen interactions at various stages of the disease progression. Finally, samples were collected from patients before and after receiving treatment to assess the impact of the treatment on immune response and pathogen expression in order to understand the effects and mechanisms of the treatment. By selecting appropriate clinical samples for testing, the pathophysiological processes of TB and the dynamic interactions between pathogens and hosts can be better understood. This helps to reveal the mechanisms of disease development and provides an important reference for the development of personalized treatment and prevention strategies.

Second, some of the published studies are based on bioinformatics analysis and have not been experimentally or clinically validated. Moreover, there is a possibility of selection bias because the data comes from public databases without the original sequencing data. In addition, because of relatively small sample sizes in some studies and some gender, age or ethnic differences, the results obtained require further validation. Additionally, the underlying mechanisms need to be further investigated. Finally, some of the studies were conducted only on animal models of *M.tb* infection and used bovine attenuated mycobacterial vaccine rather than *M.tb* to infect mice in order to create animal models for safety reasons. However, these models may not accurately represent the true clinical characteristics. Therefore, further in vivo experiments or studies using real specimens from clinical settings are necessary to obtain more accurate results.

In addition, the current single‐cell analysis of TB mainly focuses on scRNA‐seq, with a few studies combining other single‐cell sequencing technologies, such as scTCR‐seq, CITE‐seq, scATAC‐seq and so forth. However, single‐cell multi‐omics analysis includes transcriptome, genome and epigenome analysis. Single‐cell multi‐omics analysis, which encompasses transcriptome, genome, epigenome, immunome, proteome and more, offers a comprehensive and refined approach to studying TB at the single‐cell level. This strategy provides a more complete analysis for TB research. Finally, some of the potential targets discovered through single‐cell sequencing technologies have not been completely studied. Researchers can continue to explore their practical applications in the diagnosis, treatment and development of effective vaccines for TB. These targets can be combined with other scientific tools and potentially industrialized in the future to advance the real‐life clinical applications of TB research.

## 5 CONCLUSION

In this review, we provide an overview of the latest research and application of single‐cell sequencing technologies in specimens from various sources of TB. Single‐cell sequencing technologies not only analyse the composition and proportion of immune cell subpopulations, thus revealing their key roles in TB progression and the related mechanisms, but also enables the discovery of diagnostic biomarkers and therapeutic targets in TB through data analysis. Additionally, it can identify specific antigens that serve as the research basis for effective vaccine development. With the increasing application of single‐cell sequencing technology in various stages of TB, the current challenges and gaps will gradually be resolved and narrowed.

## AUTHOR CONTRIBUTIONS

**Yuqin Zeng**: Conceptualization, writing—original draft. **Quan Ma**: Writing—review & editing. **Jinyun Chen**: Writing—review & editing. **Xingxing Kong**: Writing—review & editing. **Zhanpeng Chen**: Writing—review & editing. **Huazhen Liu**: Writing—review & editing. **Lanlan Liu**: Writing—review & editing. **Yan Qian**: Writing—review & editing. **Xiaomin Wang**: Conceptualization, supervision, writing—review & editing. **Shuihua Lu**: Conceptualization, supervision, writing—review & editing. All authors read and approved the final manuscript.

## FUNDING INFORMATION

The author(s) declare financial support was received for the research, authorship and/or publication of this article. This work was supported by the Shenzhen Basic Research Special Projects (Natural Science Foundation) (No. JCYJ20220530163212027) and the National Nature Science Foundation of China (Grant Numbers 32260883 and 32394014).

## CONFLICT OF INTEREST STATEMENT

The authors declare no conflicts of interest.
